# Evaluating the Prevalence of Maternal Health Indicators on Infant Mortality Rates in Florida

**DOI:** 10.7759/cureus.63539

**Published:** 2024-06-30

**Authors:** Tamara Raymond, Jane Johnson, Shermeeka Hogans-Mathews

**Affiliations:** 1 College of Medicine, Florida State University College of Medicine, Tallahassee, USA; 2 Health Policy, Florida Association of Centers for Independent Living, Tallahassee, USA; 3 Family Medicine and Rural Health, Florida State University College of Medicine, Tallahassee, USA

**Keywords:** financial status, maternal education, prenatal care, florida counties, risk factors, infant mortality rate (imr)

## Abstract

Background

The infant mortality rate is defined as the number of infant deaths for every 1000 live births. In 2020, the infant mortality rate was 5.8% in the state of Florida, compared to 7.0% in 2000. Although infant mortality rates have declined in the state of Florida, disparities influencing these rates exist across varying Florida counties, with the widest differences being compared between the healthiest versus unhealthiest counties in Florida. Many factors can contribute to high infant mortality rates in certain counties, including income inequality, access to and quality of healthcare, race/ethnicity, obesity, and disadvantaged socioeconomic status (SES).

Methods

This study utilized data from Florida Health Charts on infant mortality rates in the state of Florida and the Pregnancy and Young Child Profiles in 10 counties to examine how certain risk factors impact infant mortality outcomes in the state of Florida. These 10 counties consist of five healthiest and five unhealthiest counties, as determined by the 2022 County Health Rankings data. T-tests were used to evaluate the relationship between county health status and several county health indicators.

Results

The average infant mortality rate from 2011 to 2020 differed significantly among healthy and unhealthy counties (p-value=0.0000). Median household income, individuals below the poverty level, and those aged zero to 17 years old were found to differ significantly by county health status (p-values 0.0000, 0.001, and 0.009, respectively). However, mothers having no high school education, births with first-trimester care, births with adequate care, and births with late or no prenatal care were not statistically significant.

Conclusion

Our study suggests that counties more likely to have fewer resources than other counties, such as those considered unhealthy, are more impacted by a higher infant mortality rate. The unhealthy counties in this study were found to have lower average median household income, higher rates of no high school education among mothers, and less prenatal care in comparison to healthy counties.

## Introduction

The infant mortality rate is defined as the number of infant deaths for every 1000 live births [1]. In 2020, the infant mortality rate was 5.8% in the state of Florida, compared to 7.0% in 2000 [2]. Although infant mortality rates have declined in the state of Florida, disparities influencing these rates exist across varying Florida counties, with the widest differences being compared between the healthiest versus unhealthiest counties in Florida. The most healthy county in Florida, St. Johns County, had an infant mortality rate of 5.0% in 2020, while the least healthy county in Florida, Union County, had an infant mortality rate of 21.4% [2,3]. Ely et al. found that infant mortality rates were higher in rural counties for infants of mothers in most age groups than in large urban counties in 2014 [4]. The social determinants of health (SDOH) are non-medical factors that influence health outcomes [5]. These factors domains include economic stability, education access and quality, health care access and quality, neighborhood and built environment, and social and community context. Many risk factors can contribute to high infant mortality rates in certain counties, including financial status, access to healthcare and physicians, race/ethnicity, and maternal education. 

Previous studies have found that one factor influencing infant mortality rate is maternal education. Infant mortality rates were shown to decline with increasing maternal education, and lowest when infants are born to mothers with a bachelor’s degree or more [6,7]. Furthermore, Ratnasiri et al. found that women who had less than a high school education were 89% more likely to experience infant deaths compared to women who had a bachelor’s degree or higher [8]. Similarly, Holland et al. found in a study of births in North Carolina that mothers with less than a high school education had the highest rate of infant mortality [9]. By contrast, infant mortality rates were significantly lower for women with higher education to the level of at least a bachelor’s degree [8]. 

When assessing the degree of association between income inequality and infant mortality in the United States, Ehntholt et al. found that there was an association at the county level but not at the state level [10]. In this study, three of seven county-level measures partially mediated this relationship, while four of seven measures mediated the association between income inequality and risk for neonatal mortality. Another study found that infants and neonates born in the United States who experienced a greater increase in income inequality since 1990 were more likely to die than those with a smaller increase in income inequality [11]. Even when adjusting for other measures of socioeconomic status (SES), Sohler et al. found that areas with higher income inequality and lower per capita income were significantly correlated with higher mortality at the zip code level [12]. These studies demonstrate how disparities in income between different areas in a state can have a substantial impact on health outcomes. 

Access to healthcare is another important factor that influences infant mortality rates. A study conducted in the Greater Newark, New Jersey area found that mothers who received no prenatal care experienced a five times greater infant mortality rate than those who had initiated prenatal care during the first trimester [13]. The timing of prenatal care can also contribute to infant mortality rates. Infant mortality rates were found to be highest for infants of women who receive late or no prenatal care [14]. When comparing racial groups among women who received prenatal care in the first trimester in this same study that analyzed data from the 2017 to 2018 National Vital Statistics System birth cohort data, infants born to Asian women had the lowest mortality (3.28%), while infants born to Black women had the highest infant mortality rate (9.64%) [6]. Infant mortality rates are uneven when comparing racial/ethnic groups, with higher rates existing for infants of Black women [8,9,14]. The infant mortality rate of Black infants was 10.8 per 1000 live births, 122% higher than the White infant mortality rate of 4.9 in 2017 [15]. 

Out of the five domains of SDOHs, our study analyzes the relationship between three SDOHs (economic stability, education access and quality, and healthcare access and quality) and the impact they may have on infant mortality rates. Although infant mortality rates have decreased in Florida over the years, notable disparities influencing these rates exist among counties considered the healthiest and unhealthiest across Florida. Few studies have examined the disparities that exist among these Florida counties given the differences in infant mortality rates. This study aimed to examine how risk factors, such as prenatal care and insurance, maternal education, and financial status, influence infant mortality rates in different counties in Florida. 

## Materials and methods

The Florida Department of Health (FDOH) maintains data on counties across the state through a community health assessment resource tool set titled Florida Health Charts. This study utilized publicly accessible data from Florida Health Charts on the 2011-2020 infant mortality rates in the state of Florida and the 2020 Pregnancy and Young Child Profiles for 10 counties [2,16]. Specifically, the 10 Florida counties analyzed in this study were the five healthiest and the five unhealthiest counties, as determined by the 2022County Health Rankings data. The County Health Rankings Roadmaps Program (CHR&R) uses a conceptual model of health that includes measures that influence each other within a county to determine a county’s ranking. These measures include health outcomes, health factors, and policies/programs. Under their model of health, policies, and programs influence health factors such as health behaviors, clinical care, social and economic factors, and the physical environment of a county. These health factors then shape the county’s health outcomes, such as length of life and quality of life [3]. The combination of these measures determines how the rankings of the Florida counties were determined by the CHR&R. 

Data analysis

According to the 2022 County Health Rankings, out of the 67 counties in the state of Florida, the top five healthiest counties are St. Johns, Collier, Seminole, Monroe, and Martin; while the lowest five unhealthiest counties are Union, Putnam, Madison, Gadsden, and Dixie (N=10). The variable “county health status” was created after indicating whether a county was determined to be healthy (coded as 1) or unhealthy (coded as 0) based on its health ranking. For statistical analyses, t-tests were used to evaluate the relationship between county health status and several county health indicators from Florida Health Charts. These county health indicators include infant mortality rate, financial status (median household income, individuals below the poverty level, and individuals below the poverty level (aged 0-17 years)), lack of high school education, prenatal care (births with first-trimester prenatal care, births with late or no prenatal care, and births with adequate prenatal care using the Kotelchuck index), and insurance status (uninsured, births covered by Medicaid, and births covered by emergency Medicaid). Data on county health indicators were utilized from the 2020 Pregnancy and Young Child Profile on each county from Florida Health Charts. These profiles list data on indicators regarding women, mothers, and births for each county in Florida. For our study, data was also collected on infant demographics and infant birth rates, female demographics, and infant mortality rates from 2011 to 2020 among the 10 Florida counties. Descriptive analyses were conducted on these variables, which were summarized by mean percentages. All analyses were performed using SAS Version 9.4 (SAS Institute Inc., Cary, NC, USA). 

## Results

Infant and female demographics

The average total percentage of births among the 10 counties was 9.45%, with the highest percentage of births being Hispanic births with an average of 13.41%. The average female population among the 10 counties was 25558 females (between ages 15 and 44), with the sample of females being predominately White females with an average of 20220. Table 1 provides mean statistics on infant and female demographics of the 10 Florida counties.

**Table 1:** Infant and female demographics

Characteristics	Mean
Infant race (%)	
Total births	9.45
White births	9.14
Black births	11.22
Other non-White births	10.25
Hispanic births	13.41
Non-Hispanic births	8.68
Female race (n)	
Total	25558.00
White	20220.20
Black	3543.90
Other	1793.90
Hispanic	6376.30
Non-Hispanic	19181.70

Infant mortality rate

Across the top five healthiest counties in Florida, the average infant mortality rate in 2020 was 4.54%, with the average infant mortality rate over the last 10 reported years (2011-2020) being 4.96%. By comparison, across the lowest five unhealthiest counties in Florida, the average infant mortality rate in 2020 was 11.57%, with the average infant mortality rate over the last 10 reported years (2011-2020) being 8.98%. The average infant mortality rate from 2011 to 2020 differed significantly among healthy and unhealthy counties (p-value=0.0000; Table 2). The infant mortality rate in 2020 was not statistically significant between healthy and unhealthy counties.

**Table 2:** County health status and infant mortality rates (N=10) ^ ***^p<0.001

Variable	Unhealthy counties (N=5) mean, SD		Healthy counties (N=5) mean, SD	T-value	P-value
Average infant mortality rate (2011-2020)	8.98 (1.08)		4.96 (0.77)	6.78***	0.000
Infant mortality rate (2020)	11.57 (6.25)		4.54 (2.30)	2.36	0.064

Financial status

The financial status of each county was analyzed by three variables: median household income, individuals below the poverty level, and individuals below the poverty level (aged 0-17 years). The average median household income between healthy and unhealthy counties in Florida was found to be statistically significant (p-value=0.0000; Table 3). Households in unhealthy counties had an average income of $42651.20, while households in healthy counties had an average income of $72430.00. Meanwhile, individuals living below the poverty level and those aged 0-17 years living below the poverty level were found to differ by county health status (p-value 0.001 and 0.009, respectively, Table 3).

**Table 3:** County health status and financial status (N=10) ^**^p<0.01 ^***^p<0.001

Variable	Unhealthy counties (N=5) mean, SD		Healthy counties (N=5) mean, SD	T-value	P-value
Median household income (in dollars)	42651.20 (7429.60)		72430.00 (6757.30)	-6.63***	0.000
Individuals below the poverty level (%)	21.34 (5.42%)		9.88 (1.30%)	4.59***	0.001
Individuals below the poverty level rate (%, aged 0-17 years)	31.88 (9.22%)		13.86 (3.74%)	4.05**	0.009

High school education

The education status of mothers in each county was analyzed by the following variable: births to mothers 19 and over without high school education. This variable was found to not be statistically significant by county health status (p=0.162; Table 4).

**Table 4:** County health status and high school education (N=10)

Variable (%)	Unhealthy counties (N=5) mean, SD		Healthy counties (N=5) mean, SD	T-value	P-value
Births to mothers 19 and over without high school education	15.62 (3.10)		10.34 (6.65)	1.61	0.162

Prenatal care and insurance

Variables examining prenatal care included the following three variables: births with first-trimester prenatal care, births with late or no prenatal care, and births with adequate prenatal care using the Kotelchuck index. Having first-trimester prenatal care, late or no prenatal care, or adequate prenatal care were all not statistically significant (Table 5). Insurance status of births included whether births came from uninsured women (self-pay checked on the birth certificate), births covered by Medicaid, and births covered by emergency Medicaid. Births that were covered by Medicaid were found to differ by county health status (p-value=0.001). However, coverage by self-pay and emergency Medicaid was not found to be statistically significant (Table 5).

**Table 5:** County health status and prenatal care and insurance (N=10) ^***^p<0.001

Variable (%)	Unhealthy counties (N=5) mean, SD		Healthy counties (N=5) mean, SD	T-value	P-value
Births with first-trimester care	71.40 (4.28)		77.24 (4.37)	-2.13	0.065
Births with late or no prenatal care	8.40 (2.68)		5.72 (1.18)	2.05	0.091
Births with adequate prenatal care	67.88 (2.89)		70.26 (6.95)	-0.71	0.509
Births to uninsured women (self-pay)	3.78 (2.02)		7.08 (5.19)	-1.33	0.241
Births covered by Medicaid	66.10 (8.60)		36.72 (9.72)	5.06***	0.001
Births covered by emergency Medicaid	1.42 (1.52)		4.02 (4.02)	-1.35	0.233

## Discussion

Our study analyzed data fromFlorida Health Charts to examine the relationship between risk factors, such as access to prenatal care and insurance, maternal education, and financial status, and their influence on infant mortality rates in different counties in the state of Florida. Our study suggests that counties that are more likely to have fewer resources than others, such as those considered unhealthy, are more impacted by a higher infant mortality rate. The unhealthy counties in this study were found to have lower average median household income, higher rates of no high school education among mothers, and less prenatal care in comparison to healthy counties. These unhealthy counties are more likely to be in rural areas and on average have a higher percentage of individuals living below the poverty level. This finding supports other studies that have found that overall infant mortality rates are highest in rural areas and lowest in large urban areas [4,17]. 

The average median household income was higher in healthy counties compared to unhealthy counties. For our sample, we found that being more financially stable was associated with living in a healthier county. This suggests that the income inequality that exists among counties poses a stronger risk factor for higher infant mortality rates at the county level. Similarly, Ehntholt et al. showed an association between county-level, but not state-level, income inequality and infant mortality [10]. That study concluded that income inequality is an important risk factor for adverse health outcomes in general. In our study, both individuals below the poverty rate and those aged 0-17 below the poverty rate had a statistically significant difference regarding county health status. Those living in unhealthy counties are more likely to have higher rates of individuals living in poverty, which can also influence high infant mortality rates for such counties. Another study also found that the infant mortality rate was directly correlated with the proportion of the population in the Greater Newark, New Jersey area living below the poverty line [13]. 

Although our study did not find a statistically significant relationship between mothers with no high school education and their county health status, our study was limited in generalizing education given the data and our small sample size. By contrast, a study conducted on infant mortality in North Carolina found mothers with less than a high school education had the highest rate of infant mortality [9]. Past research suggests that mothers who obtained a college degree or higher had a lower risk of infant mortality [8,10,14]. 

Prenatal care and health insurance for mothers are important during the prenatal period. Our study found no statistical significance for births with first-trimester prenatal care, births with adequate care, and births with late or no prenatal care. However, previous studies have found that infant mortality rates are highest for infants who received late or no prenatal care among all maternal race and Hispanic groups [6]. Infants who receive inadequate prenatal care were found to have the highest rate of infant mortality compared to those who received intermediate or adequate care [9]. To address healthcare disparities and SDOHs, such as those in this study, the American College of Physicians (ACP) recommends implementing specific policies to ensure access to high-quality care for populations at greater risk [18]. These efforts should incorporate the needs of those living in rural areas when accessing healthcare. 

However, our study was limited due to the usage of only 10 counties in Florida. This study was also limited in the use of variables provided by Florida Health Charts. Further studies should aim to include all counties in Florida to compare healthier and less healthy counties, thereby increasing generalizability through a larger sample size.

## Conclusions

The infant mortality rate in Florida has decreased over the years. Despite this decrease, counties that can be considered unhealthy are more likely to have disparities influencing high infant mortality rates. Our study suggests that these unhealthy counties are more likely to have lower household incomes, higher rates of no high school education in mothers, and less prenatal care compared to healthy counties. These results emphasize how certain risk factors can contribute to overall infant mortality rates in these areas. These results provide valuable insight into the disparities that affect counties with few resources and their impact on infant mortality rates. Addressing the SDOHs behind these disparities can have a substantial impact on people’s health. Providing expecting mothers with essential resources such as prenatal care, access to affordable healthcare, and educational resources and opportunities will help to reduce disparities and counteract negative SDOHs. In addition, access to care barriers can be decreased by increasing access and providing cost-effective services. Finally, this study shows that there is still much work to be done in improving healthcare equity in maternal and infant morbidity and mortality. 
